# Bright light therapy for depressive symptoms in hospitalized cardiac patients: A randomized controlled pilot trial

**DOI:** 10.1371/journal.pone.0230839

**Published:** 2020-03-30

**Authors:** Mark J. Eisenberg, Bettina Habib, Maria Alcaraz, Brett D. Thombs, Kristian B. Filion

**Affiliations:** 1 Center for Clinical Epidemiology, Jewish General Hospital, Lady Davis Institute, Montreal, QC, Canada; 2 Department of Medicine, McGill University, Montreal, QC, Canada; 3 Department of Epidemiology, Biostatistics and Occupational Health, McGill University, Montreal, QC, Canada; 4 Division of Cardiology, Jewish General Hospital/McGill University, Montreal, QC, Canada; 5 Departments of Psychiatry, of Psychology, and of Educational and Counselling Psychology, McGill University, Montreal, QC, Canada; Brown University, UNITED STATES

## Abstract

Depression is common among cardiac patients and associated with adverse cardiovascular outcomes. Bright light therapy has emerged as a promising treatment for depressive symptoms, however it has not yet been investigated in this population. We conducted a double-blind, randomized, placebo-controlled pilot trial to assess the feasibility of a larger-scale trial testing bright light therapy for depressive symptoms in cardiac patients. Patients hospitalized for an acute coronary syndrome or undergoing cardiac surgery were randomized to either bright light (10,000 lux) or dim light placebo (500 lux) lamps for 30 minutes each day over 4 weeks, beginning in-hospital. Depression was quantified using the Patient Health Questionnaire 9 (PHQ-9) and Depression Anxiety and Stress Scales (DASS-21). The Short-Form Health Survey 36 (SF-36) was used to measure quality of life. A total of 175 patients were screened and 15 were randomized (8 treatment, 7 placebo) (8.6%) over 10 months. Despite protocol amendments which broadened the inclusion criteria, the trial was terminated early for infeasibility based on the rate of enrollment (1–2 participants/month), with 39.5% of the target sample (38 participants) enrolled. Future trials should take into account the timing of the onset of depressive symptoms in these patients, and consider a less conservative approach to eligibility as well as ways to increase the acceptability of bright light therapy in hospitalized cardiac patients. Once enrolled, our findings suggest that most participants will adhere to the assigned treatment and complete follow-up.

## Introduction

As many as 15–20% of patients hospitalized for an acute coronary syndrome (ACS) or cardiac surgery may experience major depressive disorder [1], and many others have significant symptoms of depression [2–6]. Depression in this patient population is associated with increased cardiac mortality, re-hospitalization, recurrent myocardial infarction, repeat revascularization, and a reduced health-related quality of life [2, 3, 5–10]. Previous studies have reported on the efficacy and safety of interventions for depression such as pharmacotherapy or psychotherapy (e.g., cognitive behavioral therapy; CBT) on depressed cardiac patients. However, clinical trials and systematic reviews comparing CBT, antidepressants, and standard care only found small to moderate improvements in depression symptoms in cardiac patients [11–13]. Bright light therapy appears to reduce depressive symptoms in patients with bipolar depression [14], seasonal and non-seasonal depression [15, 16], antepartum depression [17], and in populations such as the elderly, college-aged students, and children [18–20]. However, bright light therapy has not yet been investigated in cardiac patients. We conducted a pilot trial to evaluate the feasibility of a large-scale trial testing bright light therapy among hospitalized cardiac patients.

## Methods

The “Bright Light Therapy Efficacy for Depressive Symptoms Following Cardiac Surgery or Acute Coronary Syndrome: Pilot Trial (BEAM-P)” was a double-blind, randomized, placebo-controlled pilot trial aimed at assessing the feasibility of a larger trial (clinicaltrials.gov ID: NCT02621567; https://clinicaltrials.gov/ct2/show/NCT02621567). Ethics approval was obtained from the research ethics board of the Jewish General Hospital in Montreal, Canada, prior to patient randomization. The protocol for this study and supporting CONSORT checklist are available as supporting information; see S1 CONSORT Checklist and S1 Protocol. Participants were randomized 1:1 to the bright light treatment arm or the dim light placebo arm using an online randomization service and random block sizes of 2 and 4 (randomization was not stratified). All participants provided written informed consent prior to randomization.

We included patients hospitalized for an ACS (defined as a myocardial infarction or unstable angina) or undergoing cardiac surgery (see clinicaltrials.gov for complete inclusion criteria). We excluded patients with a medical condition potentially contraindicating the use of bright light therapy, including ocular or retinal pathology, and those with conditions or taking medications increasing photosensitivity. Patients with diabetes were excluded based on their risk for eye disease (e.g., diabetic retinopathy, cataracts). The original protocol required participants to have depressive symptoms at baseline (score of ≥8 on the Patient Health Questionnaire 9 [PHQ-9]). However, this criterion was removed to increase the enrollment rate.

Participants randomized to the treatment arm received lamps that emit bright light (10,000 lux). Based on the previous literature, we elected to use 10,000 lux (the maximum lux used in previous trials) for a shorter duration (30 minutes) rather than lower lux for a longer duration (e.g., 2,500 lux for 2 hours); the longer duration of use would be more difficult for patients to adhere to and both have comparable response rates [21]. Participants in the placebo arm received lamps that emit dim light (500 lux). Although 500 lux is below therapeutic levels [21], both lamps appeared bright to participants and study staff. Participants scheduled for cardiac surgery consented to the study prior to surgery but received their lamps following surgery. Participants hospitalized for ACS received their lamps immediately following consent. All participants began lamp use while in hospital and were asked to take the lamps home once discharged.

Treatment was for a period of 4 weeks. Clinic visits took place at baseline, week 4, and week 12. A visit at the day of discharge was added to the amended protocol (to examine a potential change in depressive symptoms between prior to surgery/early hospitalization and discharge from hospital), however this visit was logistically problematic (i.e., it was often not confirmed until late in the day that the patient would be discharged), and only two participants completed the added visit. Participants were instructed to use the lamp for 30 minutes every morning, preferably within an hour of waking up and before 1 pm. They were instructed to sit 12 inches away from the lamp and to keep their eyes open for the duration of the 30 minutes, looking at a surface that the light reflected onto, but not directly into the light. Participants were allowed to use the lamps while reading, eating, or doing other seated activities.

We collected data at baseline and at follow-ups at 4 and 12 weeks. To assess depression, we used the PHQ-9 [22] and the Depression Anxiety and Stress Scales (DASS-21) [23]. Depression scores on the PHQ-9 are divided into 5 categories: normal (0–4), minimal (5–9), mild (10–14), moderate (15–19), and severe (20+). Scores on the depression subscale of the DASS-21 range from normal (0–9) to mild (10–13), moderate (14–20), severe (21–27), and extremely severe (28+). The depression scale is calculated as the sum of DASS-21 questions 3, 5, 10, 13, 16, 17, 21, multiplied by 2. In addition, the Short-Form Health Survey 36 (SF-36) was used as a self-reported measure of quality of life. SF-36 subscales include Physical Functioning, Role Functioning (physical/emotional), Vitality (energy/fatigue), Emotional Wellbeing, Social Functioning, Bodily Pain, and General Health. Numerical responses to the SF-36 are transformed according to the number of possible responses to a given question (0–6), such that the 100 is the most positive and 0 is the most negative. E.g., a question with 5 possible responses, of which the first is the most negative, is transformed as follows: 1 = 0, 2 = 25, 3 = 50, 4 = 75, 5 = 100. These responses are then averaged across all questions in a given subscale (range: 2–10 items).

Participants were asked about their use of other interventions for depression, as well as any side-effects, adverse events (AEs), or serious adverse events (SAEs) they may have experienced since the last follow-up. An AE was defined as any unfavorable or unwanted sign, symptom, or disease temporally associated with the use of the trial product, whether or not considered related to the trial product. A SAE was defined as an AE that required in-patient hospitalization or prolongation or hospitalization that resulted in persistent or significant disability or incapacity, was life-threatening, or resulted in death. Participants were asked to fill log-of-use forms every day of the 4-week treatment period to assess their adherence to protocol.

In this pilot trial, the primary endpoint was recruitment rate. Secondary endpoints included retention at week 12 and adherence through week 4 (percentage of participants that used their lamp on ≥60% of mornings within an hour of waking for at least 25 minutes). Tertiary endpoints included other aspects of feasibility (e.g., identification of challenges).

## Results

**Fig 1:**
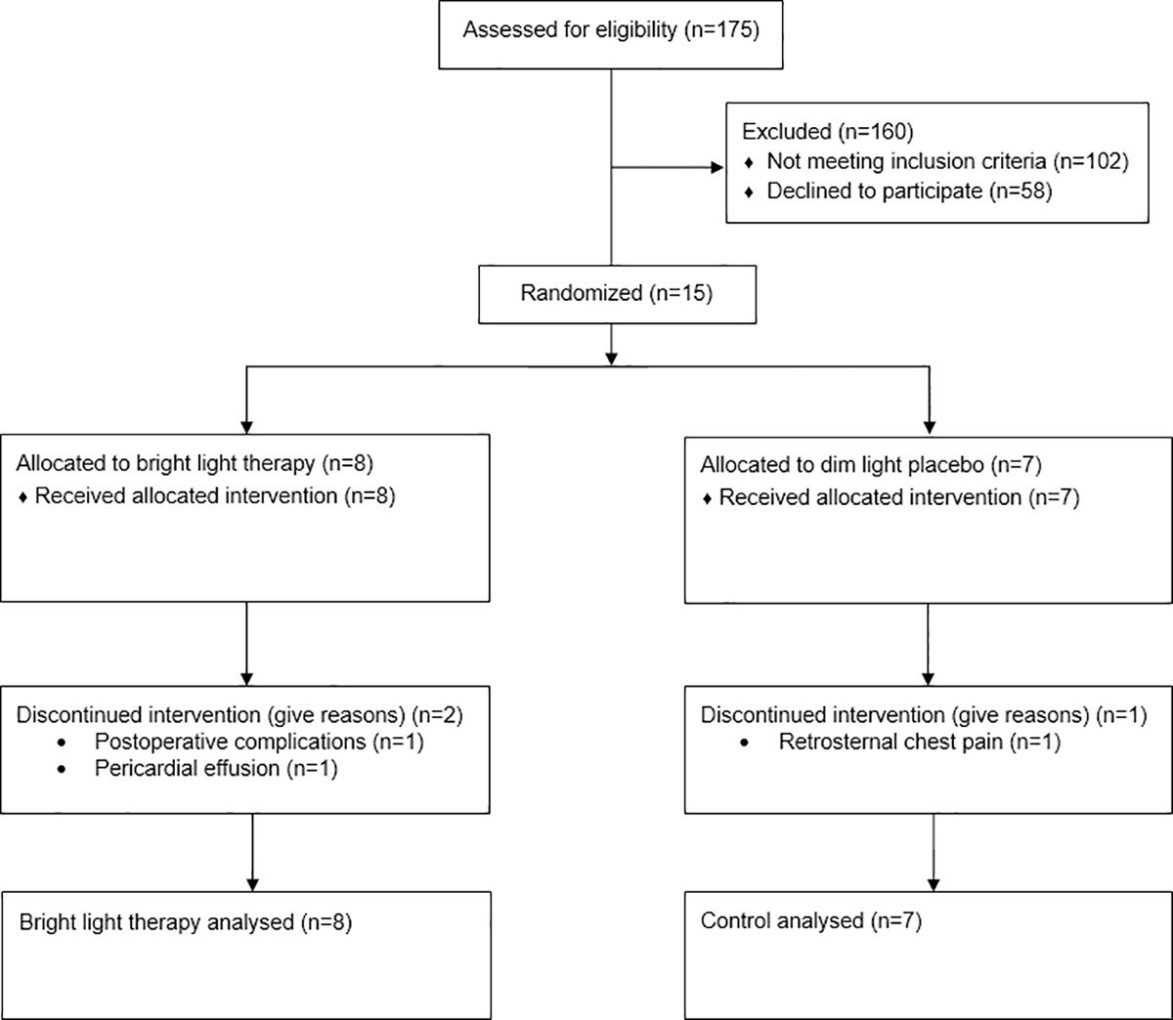
Flow chart of enrollment and follow-up in the BEAM-P trial.

**Fig 2:**
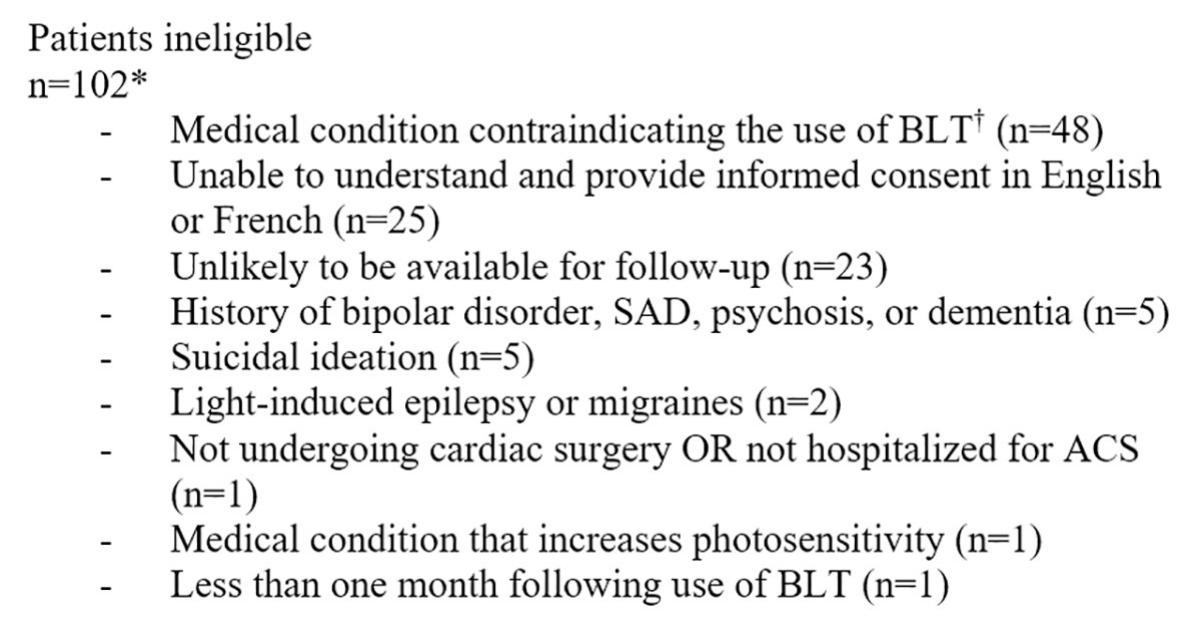
Reasons for ineligibility in the BEAM-P trial. ^*^The sum of individuals ineligible for specific reasons is >102 because several met >1 exclusion criterion. ^**†**^These include ocular or retinal pathologies such as glaucoma, cataracts, retinal detachment, and retinopathy, as well as diabetes. Abbreviations: ACS = acute coronary syndrome; BLT = bright light therapy; SAD = seasonal affective disorder.

Participants were enrolled over a period of 10 months (average of 1–2 enrollments per month). A total of 175 patients were screened (Fig 1), of whom 102 were ineligible, 58 declined to participate, and 15 were enrolled. Despite amendments to the original protocol which allowed the enrollment of patients with ACS and those without depressive symptoms at baseline, enrollment was slow and resulted in the early termination of the trial with 39.5% of the target sample (38 participants) enrolled. The prevalence of potential contraindications for bright light therapy was high in our sample. Forty-eight of the 102 (47.1%) patients deemed ineligible for the study had a medical condition cautioning against the use of bright light therapy, including ocular or retinal pathologies such as glaucoma, cataracts, retinal detachment, and retinopathy, as well as diabetes (Fig 2). However, the vast majority of eligible patients (n = 73) declined to participate (79.5%).

A total of 15 participants were enrolled in the pilot trial (Table 1). Eight (53.3%) of the enrolled participants were randomized to the bright light treatment arm, whereas 7 (46.7%) were randomized to the dim light control arm. The sample was composed of 11 men (73.3%) and 4 women (26.6%). The mean age was 60 years (standard deviation: 11). Ten participants were admitted to the hospital for an ACS, while 4 participants were admitted for coronary artery bypass grafting and one for aortic valve replacement.

**Table 1:** Participant demographics and adherence to light therapy in the BEAM-P trial.

								Depression				Lamp Use	
Pt #	Reason for admission	Age	Sex	LOS (days)	Hypertension	Cholesterol	STEMI	History of depression	Family history of depression	Use of antidepressants (current)	Smoker (current)	Days (total)	Minutes daily (average)
Treatment Arm (bright light)													
2	ACS	75	F	3	No	No	Yes	No	No	No	No	28	30
4	ACS	66	M	3	Yes	Yes	No	No	No	No	No	28	30
6	ACS	75	M	2	Yes	No	No	No	No	No	No	23	30
8	AVR	57	F	14	No	No	No	Yes	Yes	Yes	No	8^†^	30
9	CABG	53	M	10	No	Yes	No	No	No	No	Yes	6	15
12	CABG	53	M	22	No	Yes	Yes	No	No	No	No	0^§^	0
13	ACS	46	M	2	Yes	Yes	No	No	No	No	No	28	31
15	ACS	64	M	2	No	No	Yes	No	No	No	Yes	24	30
Control Arm (dim light)													
1	ACS	68	M	3	Yes	No	Yes	No	No	No	No	28	62
3	CABG	77	M	9	Yes	No	No	No	No	No	No	24	32
5	ACS	56	M	2	No	Yes	No	No	No	No	No	24	30
7	ACS	60	M	4	Yes	Yes	No	Yes	Yes	Yes	Yes	19	30
10	CABG	65	F	12	No	Yes	No	No	No	No	No	27	30
11	ACS	37	F	3	No	No	No	No	No	No	Yes	24	33
14	ACS	59	M	4	No	No	No	No	No	No	No	0^§^	0

Abbreviations: ACS, Acute Coronary Syndrome; CABG, Coronary Artery Bypass Graft; AVR, Aortic Valve Replacement; LOS, Length of Stay; Pt, participant

†Participant discontinued lamp use only one week after use due to fatigue and re-hospitalization.

^§^ Following enrolment, participant decided not to use lamp at all but agreed to remain in the study for follow-ups.

Most participants (73.3%) were adherent to therapy (use on ≥60% of mornings for at least 25 minutes). Participants used their lamps for a median of 24 days, interquartile range (IQR: 23.8, 28) for a median of 30 minutes (IQR: 30, 30.5) per day over the course of the 28 day treatment period. Of the 15 participants, 2 participants (one from each treatment arm) decided not to use the lamp after enrollment, prior to any lamp use. One additional participant from the treatment arm discontinued use after one week due to fatigue and re-hospitalization. All participants completed the week 4 and week 12 follow-ups (100% retention).

This pilot trial was not powered to detect differences in depressive symptoms or quality of life between the bright light therapy and dim light therapy arms. According to the PHQ-9 and DASS-21 depression subscale, no participant had more than mild depressive symptoms at baseline (PHQ-9 scores ranged from: 0–13; DASS-21 scores ranged from: 0–12) (Table 2). Quality of life varied widely between participants and among the eight SF-36 subscales (Table 3). Summary measures are provided in Table 4.

**Table 2:** Participant PHQ-9 and DASS-21 depression subscale scores in the BEAM-P trial.

	PHQ-9			DASS-21*		
Pt #	Baseline	Week 4	Week 12	Baseline	Week 4	Week 12
Treatment Arm (bright light)						
2	4	5	9	2	2	6
4	8	2	4	6	0	0
6	3	0	1	0	0	0
8	11	20	13	0	32	18
9	13	6	9	10	4	4
12	3	15	8	2	18	14
13	4	0	0	0	0	0
15	2	3	6	0	2	6
Control Arm (dim light)						
1	0	0	1	0	0	0
3	0	0	0	0	0	0
5	2	1	2	10	0	0
7	3	2	0	10	4	4
10	2	12	0	12	16	14
11	11	5	2	10	4	4
14	2	10	16	0	12	28

Pt, participant; PHQ-9, Patient Health Questionnaire 9; DASS-21, Depression, Anxiety, and Stress Scale 21

* Depression scale (sum of questions 3, 5, 10, 13, 16, 17, 21, multiplied by 2)

**Table 3:** Participant SF-36 scores in the BEAM-P trial*.

	Physical Functioning			Role Functioning (P)			Role Functioning (E)			Energy/Fatigue			Emotional Wellbeing			Social Functioning			Bodily Pain			General Health		
Pt #	Wk 0	Wk 4	Wk 12	Wk 0	Wk 4	Wk 12	Wk 0	Wk 4	Wk 12	Wk 0	Wk 4	Wk 12	Wk 0	Wk 4	Wk 12	Wk 0	Wk 4	Wk 12	Wk 0	Wk 4	Wk 12	Wk 0	Wk 4	Wk 12
Treatment Arm (bright light)																								
2	70	80	45	75	0	0	0	0	0	30	60	50	80	68	60	38	50	63	68	88	100	55	65	45
4	40	75	70	0	75	0	100	100	100	55	50	55	72	80	80	NR^	100	88	45	68	68	35	50	40
6	80	85	90	100	75	100	100	100	100	80	70	70	92	92	92	88	88	100	55	80	90	70	60	60
8	60	10	35	0	0	0	0	0	0	15	5	5	52	36	36	50	0	38	10	20	45	60	40	30
9	50	85	85	25	0	25	0	0	100	60	50	45	72	76	88	63	63	75	78	78	65	45	60	65
12	25	25	90	100	0	0	100	0	0	70	50	60	72	48	48	75	50	38	55	68	78	40	40	50
13	65	80	60	50	100	100	67	100	100	75	90	65	96	100	96	100	100	100	80	68	58	40	70	55
15	90	80	72	100	25	100	100	100	67	55	65	50	64	72	52	100	100	63	78	90	90	70	65	60
Control Arm (dim light)																								
1	95	100	95	100	100	100	100	100	100	75	70	70	80	80	88	100	100	100	68	78	68	95	85	90
3	65	65	100	0	0	100	100	100	100	100	100	100	100	100	100	100	100	100	38	80	100	100	100	100
5	60	90	100	100	25	100	100	100	100	70	65	70	92	88	96	100	75	100	100	100	100	70	80	80
7	35	55	60	0	25	0	0	33	100	40	60	45	72	92	84	38	50	50	33	68	45	20	15	15
10	55	45	75	25	0	25	100	100	67	50	25	40	56	68	36	88	50	75	100	10	68	90	85	75
11	15	85	95	0	100	100	100	67	67	25	70	75	64	84	92	25	75	75	23	80	100	65	69	90
14	95	65	10	50	0	0	100	0	0	50	15	0	68	68	20	75	50	25	38	58	23	80	75	30

Pt, participant; SF-36, Short Form (36) Health Survey; Wk 0, baseline

^Participant did not answer either of questions 20 and 32, which make up the entirety of the social functioning subscale.

*Numerical responses to the SF-36 are transformed according to the number of possible responses to a given question (0–6), such that the 100 is the most positive and 0 is the most negative. E.g., a question with 5 possible responses, of which the first is the most negative, is transformed as follows: 1 = 0, 2 = 25, 3 = 50, 4 = 75, 5 = 100. These responses are then averaged across all questions in a given subscale (range: 2–10 items).

**Table 4:** Summary scores on the PHQ-9, DASS-21 depression subscale, and SF-36 by intervention group in the BEAM-P trial.

		Treatment Arm (Bright Light) n = 8			Control Arm (Dim Light) n = 7		
		Baseline	Week 4	Week 12	Baseline	Week 4	Week 12
**PHQ-9, Median (IQR)**		4 (3, 8.8)	4.0 (1.5, 8.3)	7 (3.2, 9)	2 (1, 2.5)	2 (0.5, 7.5)	1 (0, 2)
	Normal (0–4) (%)	62.5	50	37.5	85.7	57.1	85.7
	Minimal (5–9) (%)	12.5	25	50	0	14.3	0
	Mild (10–14) (%)	25	0	12.5	14.3	28.6	0
	Moderate (15–19) (%)	0	12.5	0	0	0	14.3
	Severe (20+) (%)	0	12.5	0	0	0	0
DASS-21*, Median (IQR)*		1 (0, 10)	2.0 (0, 7.5)	5 (0, 8)	4 (0.5, 9)	4.0 (0, 8)	4.0 (0, 9)
	Normal (0–9) (%)	87.5	75	75	42.8	71.4	71.4
	Mild (10–13) (%)	12.5	0	0	57.1	14.2	0
	Moderate (14–20) (%)	0	12.5	25	0	14.2	14.2
	Severe (21–27) (%)	0	0	0	0	0	0
	Extremely Severe (28+) (%)	0	12.5	0	0	0	14.2
**SF-36, Median (IQR)**							
	Physical Functioning	63 (48,73)	80 (63, 81)	71 (56, 86)	60 (45, 80)	65 (60, 88)	95 (68, 98)
	Role Functioning (P)	63 (19, 100)	13 (0, 75)	13 (0, 100)	25 (0, 75)	25 (0, 63)	100 (13, 100)
	Role Functioning (E)	83 (0, 100)	50 (0, 100)	83 (0, 100)	100 (100, 100)	100 (50, 100)	100 (67, 100)
	Energy/Fatigue	58 (49, 71)	55 (50, 66)	53 (49, 61)	50 (45, 73)	65 (43, 70)	70 (43, 73)
	Emotional Wellbeing	72 (70, 83)	74 (63, 83)	70 (51, 89)	72 (66, 86)	84 (74, 90)	88 (60, 94)
	Social Functioning	75 (56, 94)	75 (50, 100)	69 (56, 91)	88 (56, 100)	75 (50, 88)	75 (63, 100)
	Bodily Pain	61 (53, 78)	73 (68, 82)	73 (63, 90)	38 (35, 84)	78 (63, 80)	68 (56, 100)
	General Health	50 (40, 63)	60 (48, 65)	53 (44, 60)	80 (68, 93)	80 (72, 85)	80 (53, 90)

PHQ-9, Patient Health Questionnaire 9; DASS-21, Depression, Anxiety, and Stress Scale 21; SF-36, Short Form (36) Health Survey; Role Functioning (P), Physical role functioning limitations; Role Functioning (E), Emotional role functioning limitations.

*Depression subscale

Three participants in total (20%) scored above “mild” on the PHQ-9 or above “moderate” on the DASS-21 at least once during follow-up. These were also the only participants who reported experiencing a SAE during the 12 week follow-up period. Participant number 8, from the bright light arm, suffered from pericardial effusion and discontinued lamp use after 8 days. This participant scored “severe” and “extremely severe” on the PHQ-9 and DASS-21, respectively, at week 4. Participant number 12, from the treatment arm, suffered from postoperative complications and did not use the lamp. This participant scored “moderate” on the PHQ-9 at week 4. Finally, participant number 14, from the control arm, suffered from retrosternal chest pain and did not use the lamp. This participant scored “moderate” on the PHQ-9 and “extremely severe” on the DASS-21 at week 12. All 3 participants also had relatively lower scores than others on the Emotional Wellbeing subscale of the SF-36 (lower scores on this scale indicate lower health). All SAEs were considered unrelated to the use of the lamps.

## Discussion

Our pilot study was designed to assess the feasibility of a large-scale trial of bright light therapy for depression in hospitalized cardiac patients. The trial was terminated early for slow enrollment after 10 months, with 15 participants out the target sample size of 38 enrolled. Nearly 60% of screened patients were ineligible, and 4 out of every 5 eligible patients refused to participate. No participant had more than mild depressive symptoms at baseline. However, enrolled participants were largely adherent to their assigned treatment, and retention in the study was 100% at the end of follow-up. Overall, the findings from this small, single center pilot trial suggest that a large-scale trial would be challenging to conduct.

Several trials have investigated the potential of bright light therapy as a treatment modality for non-seasonal depression. In 2005, Golden et al. [21] conducted a systematic review and meta-analysis of the efficacy of bright light therapy in the treatment of mood disorders. Twenty randomized controlled trials were included in the analysis. The meta-analysis demonstrated significant effects of bright light therapy for the treatment of seasonal affective disorder and non-seasonal depression. Another review conducted in 2004 [24] of 49 studies found that, in high-quality studies, the response to bright light was significantly better than the response to control interventions.

The results of our feasibility study suggest that it would be difficult to study the use of bright light therapy for the treatment of depression following hospitalization for ACS or cardiac surgery. Very few patients scored higher than “normal” on either depression scale at any time during the study. Our original protocol included only patients with a baseline PHQ-9 score ≥8. If the protocol had not been amended to remove this criterion, only 4 participants would have been enrolled. This suggests that future trials need to consider the timing of the occurrence of depressive symptoms following a cardiac hospitalization, in order to determine the optimal timing of interventions to treat depression. Likewise, it remains unclear which patients would benefit the most from bright light therapy.

Large-scale studies could consider a less conservative approach to eligibility. As a preventative measure, experts recommend that patients with retinal diseases avoid bright lights [25]. Therefore, we excluded patients with diabetes based on their elevated risk for diabetic retinopathy and cataracts, (47.1% of ineligible patients had diabetes, cataracts, or both). However, it is not clear whether diabetes, in the absence of retinopathy, constitutes a contraindication for bright light therapy. In addition, the literature about the potential adverse effects of light therapy on the eye is inconclusive. A study by Gallin et al. examined the effects of 30 minutes of daily exposure to 10,000 lux in 50 patients with seasonal depressive disorder for a period of up to 6 years of use and detected no ocular changes in the patients [26]. Even with regards to patients with retinopathy, outdoor exposure to sunlight is considered more hazardous than short-term exposure to bright light therapy [27]. It seems likely that a larger trial could safely enroll patients with diabetes who do not have diabetic retinopathy.

Future studies should also consider the acceptability of bright light therapy in patients with cardiovascular disease. Refusals to participate among eligible patients were high, however the reasons for refusal were not recorded. It seems plausible that patients who are have poorer physical and/or mental health were less likely to participate. Among our enrolled participants, the only patients who reported higher than “normal” depression scores were the same patients that experienced SAEs and discontinued lamp use before the end of the treatment period. It is possible that sustained health problems in these patients caused higher depressive symptoms and lower adherence to the study. If this were the case, the importance of adherence would need to be reinforced for bright light therapy to be effective in this population. Trials evaluating the efficacy of bright light therapy for non-seasonal depression in patients following cardiac hospitalization should also take seasonality into account. A questionnaire such as the Seasonal Pattern Assessment Questionnaire (SPAQ) could be used as part of a baseline assessment, and adjusted for in analyses of efficacy.

## Conclusions

This pilot study was designed to evaluate the feasibility of a larger-scale trial testing the efficacy of bright light therapy for depressive symptoms among hospitalized cardiac patients. Overall, the findings from this small, single center trial suggest that a large-scale trial would be challenging to conduct. Future trial designs should take into account the timing of the onset of depressive symptoms around hospitalization and the impact of seasonality in these patients. Researchers should also consider a less conservative approach to eligibility (e.g., including patients with diabetes), as well as ways to increase the acceptability of bright light therapy in hospitalized cardiac patients. Enrollment may be feasible if these factors are addressed. Once enrolled, our findings suggest that most participants will adhere to the assigned treatment and complete follow-up.

## Supporting information

S1 CONSORT Checklist (DOC)

S1 Protocol (Original). (PDF)

S2 Protocol (Amended). (PDF)
