# Supplementary material for: Bright light therapy for depressive symptoms in hospitalized cardiac patients: A randomized controlled pilot trial
Source: PLoS One. 2020 Mar 30;15(3):e0230839. doi: 10.1371/journal.pone.0230839 (PMC7105123; doi:10.1371/journal.pone.0230839)
Supplement: S1 Protocol — (PDF) [file pone.0230839.s002.pdf]

Bright Light Therapy Efficacy for Depressive Symptoms Following Cardiac Surgery: Pilot Trial (BEAM-P)

Sponsor-Investigator: Mark J. Eisenberg, MD MPH (Jewish General Hospital, Montreal, QC)

Divisions of Cardiology and Clinical Epidemiology
Jewish General Hospital/McGill University
3755 Côte-Sainte-Catherine Rd/Suite H-421
Montréal, Québec, H3S 1Y9, Canada
Tel: (514) 340-8222 x3564
Fax: (514) 340-7564
E-mail: mark.eisenberg@mcgill.ca

Funding Organization: LDI Clinical Research Pilot Project (CliPP) Funding Initiative

Protocol Number: BEAM-P-01

Date of Protocol: November 17 2015

SIGNATURE PAGE

Sponsor-Investigator

I have reviewed this clinical trial protocol and confirm this document is correct and complete.

I have read and understand the requirements for this protocol. I confirm my agreement to conduct the trial in compliance with the protocol.

I acknowledge that I am responsible for the overall conduct of this trial at my site. I agree to personally conduct and/or supervise the described clinical trial and to comply with its requirements, subject to ethical and safety considerations and guidelines, and to conduct the trial in accordance with the International Conference on Harmonization guidelines on Good Clinical Practice (ICH-GCP) and applicable Health Canada requirements.

_______________________________ 11/19/15

Mark J. Eisenberg, MD MPH
Professor of Medicine
Divisions of Cardiology and Clinical Epidemiology
Jewish General Hospital/McGill University
3755 Côte-Sainte-Catherine Rd/Suite H-421
Montréal, Québec, H3S 1Y9, Canada
Tel: (514) 340-8222 x3564
Fax: (514) 340-7564
E-mail: mark.eisenberg@mcgill.ca

Date

TABLE OF CONTENTS

**Section**	**Page**
Protocol Synopsis	5
1. Trial Objectives and Purpose	6
1.1 Overall Trial Objective	6
1.2 Primary Objectives	6
1.3 Rationale	6
2. Background Information	6
2.1 Summary of Previous Findings	6
3. Investigational Products	8
3.1 Name of Investigational Products	8
3.2 Description of Investigational Products	8
3.3 Route of Administration	8
3.4 Dose and Dosage Regimen	8
3.5 Product Accountability	8
3.6 Treatment Period	8
3.7 Discontinuation Criteria	9
3.8 Disallowed Medications	9
3.9 Potential Risk	9
3.10 Potential Benefits	9
4. Trial Design	9
4.1 Summary of Trial Design	9
4.2 Schematic Diagram of Trial Design	10
4.3 Primary Endpoint	10
4.4 Secondary Endpoints	10
4.5 Tertiary Endpoints	11
4.6 Measures Taken to Reduce Bias	11
4.6.1 Randomization	11
4.6.2 Blinding	11
4.7 Study Population	11
4.8 Inclusion and Exclusion Criteria	11
4.8.1 Inclusion Criteria	11
4.8.2 Exclusion Criteria	11
5. Trial Procedures	12
5.1 Recruitment	12
5.2 Enrollment	12
5.3 Treatment Arms	12
5.3.1 Randomization to Treatment	12
5.3.2 Treatment	12

5.4 Follow-up	13
5.4.1 Duration of Participation	13
5.4.2 Data Collection	13
5.4.3 Source Data	13
5.4.4 Schedule of Events	13
5.4.5 Loss to Follow-up	14
5.4.6 Participant Withdrawal	14
5.4.7 Confidentiality	15
6. Trial Safety	15
6.1 Collection of Safety Data	15
6.2 Adverse Events	15
6.3 Serious Adverse Events	15
6.4 Unblinding	15
7. Data Analyses	16
7.1 Description of Data Analyses	16
7.3 Sample Size Justification	16
References	17

PROTOCOL SYNOPSIS

Title of Trial:	Bright Light Therapy Efficacy for Depressive Symptoms Following Cardiac Surgery: Pilot Trial (BEAM-P)
Short Title of Trial:	BEAM-P
Sponsor-Investigator:	Mark J. Eisenberg, MD MPH (Jewish General Hospital, Montreal, QC)
Trial Sites:	Single centre
Trial Phase:	IV
Primary Objective:	(1) To assess the feasibility of conducting a full efficacy trial.
Methodology:	Randomized (1:1), controlled trial
Sample Size:	38 participants
Study Population:	Cardiac surgery patients experiencing moderately depressive symptoms.
Trial Arms:	(1) Bright light therapy lamp (10,000 lux light intensity); (2) Modified dim lamp (500 lux)
Treatment Duration:	4 weeks
Follow-up Duration:	12 weeks (inclusive of the 4 week treatment period)

1. TRIAL OBJECTIVES AND PURPOSE

1.1 Overall Trial Objective

The Bright Light Therapy Efficacy for Depressive Symptoms Following Cardiac Surgery: Pilot Trial (BEAM-P) is a randomized controlled trial (RCT) that seeks to assess the feasibility of conducting a full trial which would assess the efficacy of Bright Light Therapy (BLT) for improving depressive symptoms post-cardiac surgery.

1.2 Primary Objective

To assess the feasibility of conducting a full trial, including assessment of recruitment rate, retention rate, adherence, process time, and potential additional data values that should be measured.

1.3 Rationale

More than 50,000 Canadians undergo cardiac surgery each year;[1,2] and of these patients, at least 15-20% experience depression.[2,3] The rate of depression in this population is almost three-fold greater than in the general population, and tends to be persistent.[2] Unfortunately, depression remains under recognized and undertreated.[4] Depression has widespread health, economic, and social consequences.[5] A study conducted in Quebec found that the costs of treating myocardial infarction (MI) patients who are depressed are 41% higher than for non-depressed patients.[6] Treating depressive symptoms in cardiovascular patients can substantially contribute to reducing healthcare expenses. Cardiovascular patients with depression have also been found to be at greater risk of morbidity and mortality than non-depressed cardiovascular patients (hazard ratio 2.2).[7] Risk of mortality in MI patients with depressive symptoms is significantly increased in the first 6 and 18 months post-MI.[8] Even mild depressive symptoms (not considered clinically significant) have been shown to increase the risk of death after an acute MI.[9] Depressive symptoms in cardiac patients have severe consequences and must be adequately treated in order to reduce the risk of morbidity and mortality. Novel efficacious treatments for depressive symptoms are urgently needed.

BLT may represent an innovative and effective method for reducing depressive symptoms in cardiac surgery patients. BLT has previously been shown to decrease depressive symptoms in individuals with seasonal and non-seasonal depression.[10,11] A Cochrane review of BLT for non-seasonal depression concluded that BLT was significantly more effective at reducing depression than placebo and is a promising therapy for non-seasonal depression.[12] BLT for non-seasonal depression has been shown to produce benefits in the range of 12-35% reduction in depressive symptoms, often within a week of starting treatment.[13] BEAM-P will be the first trial to study the effects of BLT on depressive symptoms in cardiovascular patients. Since BLT has not yet been investigated in cardiac patients, the feasibility of conducting a large trial of BLT in this population must be determined in order to avoid potential pitfalls and enhance the chances of success of a full trial.

2. BACKGROUND INFORMATION

2.1 Summary of Previous Findings

We conducted a non-systematic search of PubMed to identify studies of BLT for non-seasonal depression. We did not identify any studies of BLT for depression in cardiac surgery patients.

However, we found which investigated BLT for depression in other patient populations, such as elderly patients, adolescents, pregnant women, and epilepsy patients. These previous studies generally had a number of serious limitations, including small sample sizes, short treatment and follow-up periods, and inappropriate controls. Blinding was an issue in some studies because differently colored lights (blue, red, green, white) were used.[12] The studies with fewer limitations are described below.

One double-blind RCT investigated the effects of BLT for elderly patients with non-seasonal major depressive disorder.[11] Efficacy was assessed using mean improvement on the Hamilton Rating Scale for Depression scores from baseline to the end of the treatment period (week 3), and to the end of follow-up (week 6). They recruited 89 participants and randomized them to 3 weeks of daily BLT (60 minutes, blue light, 7,500 lux), or placebo (dim red light, 50 lux). Analyses showed significantly greater improvement in the BLT group compared to placebo from baseline to week 3 (43% vs 36%). This improvement was maintained during follow-up (54% vs 33%). BLT also significantly decreased cortisol levels (34% decrease), an indicator of stress, compared to placebo (7% increase). The authors concluded that BLT was effective at reducing depressive symptoms compared to placebo.

A randomized crossover trial took place to investigate the potential of BLT to reduce depressive symptoms in mildly depressed adolescents.[14] After a week of baseline measurements, 28 participants were randomized to receive the therapeutic intervention or the control intervention; half of participants got BLT (2,500 lux) first while half received the dim light (50 lux) first. After 1 week, the participants switched and received the other intervention. The Beck Depression Inventory was used to measure depressive symptoms every week during the trial, and at 1 week post-treatment. The Wilks Lambda test was performed and suggested that there were significant differences between Beck Depression Inventory scores due to the BLT treatment ($F=3.92$, $p=0.014$).

A double-blind RCT for antepartum depression was performed to determine the effects of BLT compared to dim light.[15] For 1 hour per day for 5 weeks, 27 participants used a white light (7,000 lux) or a dim red light (70 lux), depending on group allocation. Depressive symptoms were measured using the Hamilton Depression Rating Scale each week. Analyses showed that BLT treatment was significantly more effective at reducing depressive symptoms compared to the dim light group (effect size 0.78). Significantly more individuals in the BLT group than the control group achieved remission from depression (69% vs 36%).

Finally, a RCT investigated the effect of BLT on symptoms of anxiety and depression.[16] This double-blind study enrolled 101 adults with focal epilepsy. After 12 weeks of being followed, participants received the intervention for 12 weeks: BLT at 10,000 lux for 20-30 minutes per day, or dim light at 2,000 lux for 20-30 minutes per day. Anxiety and depression were measured using the Hospital Anxiety and Depression Scale. No difference in anxiety was found from baseline to end of treatment between the groups. Both groups significantly improved from baseline to end of treatment in depression score. This result may be explained by the fact that 2000 lux (the placebo intervention) has been used as a therapeutic intervention in past studies, and has been shown to be effective.[14,17,18] Although 101 participants started this trial, only 58 remained by the end, resulting in a high loss to follow-up rate of 42%. This may be explained in part by the investigators recruiting all patients, regardless of depression status (resulting in less incentive to undergo treatment and remain in the trial).

More rigorous, controlled studies are needed to investigate BLT's potential for reducing depressive symptoms in different patient populations. The studies described above had various limitations which the full BEAM Trial will improve upon. We will ensure proper blinding (both lamps will have white light that appears bright), adequate treatment and follow up periods (4, and 52 weeks, respectively), and sufficient sample size. Research investigating BLT for depressive symptoms in cardiac surgery patients is crucial in order to improve patient morbidity and mortality outcomes.

3. INVESTIGATIONAL PRODUCTS

3.1 Name of Investigational Products

The BEAM-P Trial will use BLT lamps that emit 10,000 lux, and lamps modified to emit 500 lux. These lamps are the TRAVelite lamps manufactured by Northern Light Technologies.

3.2 Description of Investigational Products

We will be using the TRAVelite Desk Lamp manufactured by Northern Light Technologies, located in Montreal, Quebec. The light is emitted from a lamp. The florescent tubes in the box are covered with a diffusing screen to ensure even distribution of light and protection from ultraviolet light.[19] Half of the lamps will emit 10,000 lux, while the rest will be modified to emit 500 lux. Both lamps will look identical and the difference in the light emitted will only be apparent if both lamps are placed next to each other. This will ensure that neither the study personnel nor the participants are aware of the participants' random allocation. These lamps have a diffusing screen covering the bulbs to evenly diffuse the light and protect against ultraviolet rays. These lamps also have a stand which enables users to place the box horizontally or vertically on most surfaces. BLT lamps can be purchased by the public at various stores, including many pharmacies.

3.3 Route of Administration

Participants will be exposed to the light by sitting 12 inches from the lamp while looking at a surface that the light is reflecting onto. They should not look directly into the light, but may glance at it occasionally. This therapy can be done while reading, eating, or doing other seated activities.

3.4 Dose and Dosage Regimen

Participants will be instructed to use the light for 30 minutes each morning within an hour of waking, but not past 1:00 pm (to ensure that sleep does not become disturbed). The light must be approximately 12 inches from the participants' faces. Rulers will be used in hospital to ensure participants use the lamp at the proper distance. The length of time, and distance from the lamp both affect the intensity of light being received. Participants' eyes should be open when using the lamp, but they should not look directly into the lamp.

3.5 Product Accountability

All lamps will be blinded using blind codes. An inventory sheet listing the blind codes will be retained. The lamps will be stored in a locked cabinet inside the investigator's office. An accountability log will be kept to log dispensing of lamps to enrolled participants.

3.6 Treatment Period

The treatment period will last 4 weeks and will begin once the participant consents to be in the study, is found to be eligible, and is randomized. The treatment will begin in-hospital and will continue at home after hospital discharge, for the remainder of the 4 weeks.

3.7 Discontinuation Criteria

Discontinuation of lamp use due to participant preference is permitted. These participants will be requested to continue with study follow-ups as per protocol. No negative consequences are anticipated from abrupt discontinuation of the investigational product. Use of the investigational product should be discontinued if participants experience severe or worsening symptoms: eye pain, photosensitivity, suicidal ideation.

3.8 Disallowed Medications

During the 4 week treatment period, participants will be allowed to receive other treatments for depression (standard of care); use of such therapies will be recorded.

3.9 Potential Risks

We do not anticipate any major safety concerns with the use of BLT. All BLT lamps will be cleaned and sterilized before being given to participants, as well as when participants leave the hospital, to prevent possible spread of infection and reduce contamination concerns. The side effects that have been reported with BLT include headache, eyestrain, irritability/agitation, mania associated with bipolar disorder, and blurred vision or nausea, but are typically mild and disappear after the first few days.[10,20] Participants have rarely dropped out of studies due to BLT side effects.[21]

3.10 Potential Benefits

Light therapy was first introduced in 1984 as a treatment for Seasonal Affective Disorder.[20] It has proven to be quite effective at reducing depression in this population.[19,20,22,23] More recently, BLT has been marketed and studied for all types of depression, as well as for mood, energy, fatigue, alertness, focus, and concentration.[24,25] BLT has been shown to reduce depressive symptoms in patients with seasonal depression as well as non-seasonal depression or depressive symptoms.[10-12,14,16,17,20,22,23,26] Some studies have also found BLT to improve anxiety, sleepiness, fatigue, sleep efficiency, and health-related quality of life.[11,16,22]

4. TRIAL DESIGN

4.1 Summary of Trial Design

We will conduct a RCT with a treatment period of 4 weeks and 12 week follow-up post-cardiac surgery. A total of 38 cardiac surgery participants will be randomized 1:1 to one of two treatment arms: 1) BLT or 2) Dim light (control group). Participants randomized to the BLT arm will receive a lamp emitting 10,000 lux light intensity, while those in the dim light arm will receive lamps emitting 500 lux. These lamps will both appear bright to participants, but only the high intensity light has been shown to be therapeutic.[10] We will be using the TRAVelite Desk Lamp manufactured by Northern Light Technologies. Study personnel and participants will be blinded as to treatment allocation. Permutated block randomization will help ensure a balance of known and unknown confounders. Informed consent will be obtained in person from all individuals. Potential participants will be informed that the purpose of this pilot study is to assess the feasibility of carrying out a large trial to investigate BLT for depressive symptoms in cardiac surgery patients. The follow-up visits and data to be collected will be discussed, as well as alternatives to participation, issues of confidentiality, and

participants' rights and responsibilities. Potential participants will be given sufficient time to ask questions and decide whether or not to participate prior to signing and receiving a copy of the signed consent form.

Participants will also be provided with an information sheet containing study procedures and contact information for study personnel. Participants in both groups will begin using the lamps in-hospital and will continue the treatment at home for the remainder of the 4 weeks post-surgery. At baseline, participants will complete the Patient Health Questionnaire 9 (PHQ-9), Depression Anxiety Stress Scale short version (DASS-21), and Short Form (36) Health Survey (SF-36) questionnaires in order to determine index levels of depressive symptomology (PHQ-9, DASS-21) and health-related quality of life (SF-36). A case report form, to collect demographic and clinical information, will be completed at baseline. On day 4 and on the day of discharge, participants will complete questionnaires and a CRF will be completed assessing adherence, tolerability, and side effects. There will be a clinic visit at week 4, when patients will return their lamp and complete questionnaires, and study personnel will complete a case report form to monitor treatment adherence. At week 12, participants will have the option to complete their questionnaires online or by mail. To compensate participants for expenses incurred due to participation (parking, childcare, opportunity costs, etc.), they will receive $25 at week 4 and a $20 prepaid VISA card after completing the questionnaires at week 12.

4.2 Schematic Diagram of Trial Design

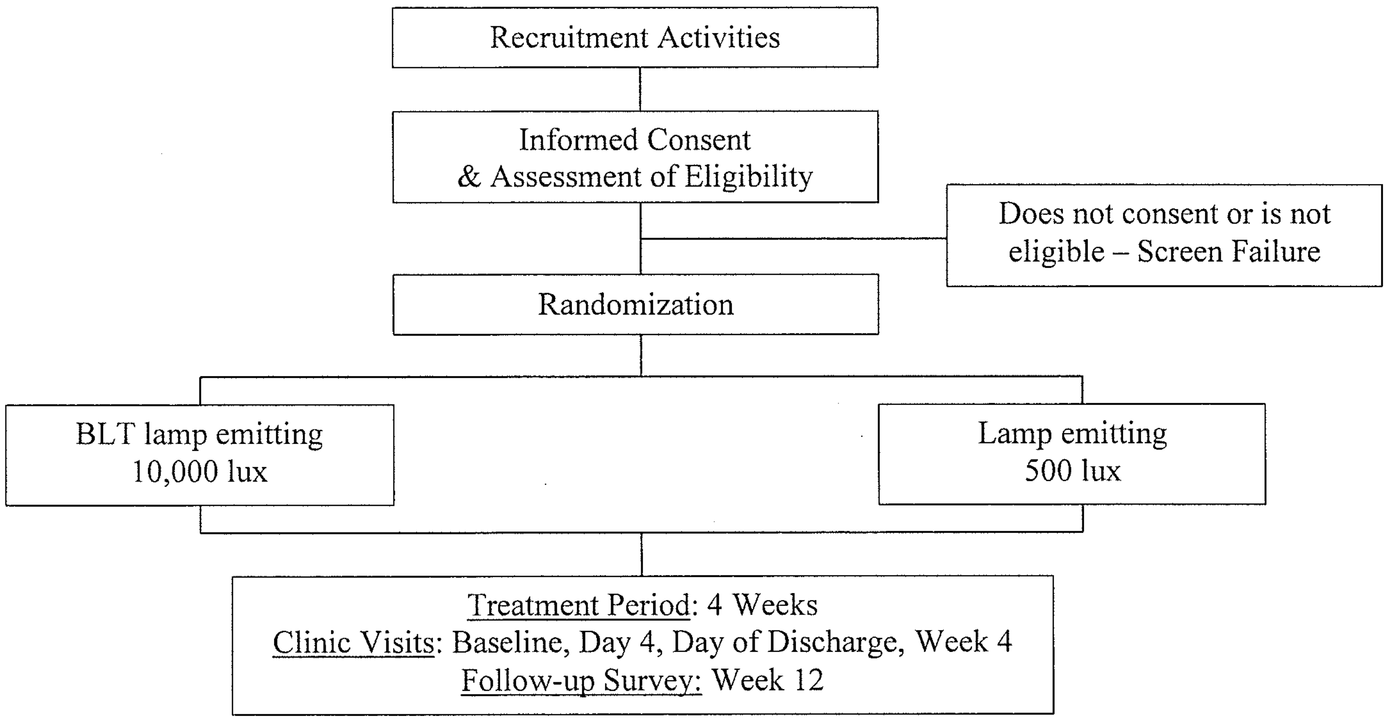

4.3 Primary Endpoint

(1) Recruitment rate

4.4 Secondary Endpoints

(2) Retention rate: percentage of participants who remain in the study at week 12.

(3) Adherence rate: percentage of participants that used their lamp on $\geq$60% of mornings within an hour of waking for at least 25 minutes.

4.5 Tertiary Endpoints

(4) Determination of process time required
(5) Identification of unforeseen challenges
(6) Identification of important data values that were not measured

4.6 Measures Taken to Reduce Bias

4.6.1 Randomization

The use of randomization should result in a balance of known and unknown confounders. We will use stratified, permuted block randomization. A permuted block design will prevent study personnel from identifying the randomization sequence.

4.6.2 Blinding

Randomization to the therapy lamps will be blinded as to whether they emit 10,000 lux or 500 lux. The lamps are identical in appearance when turned off. To prevent study personnel unblinding, participants will be asked to turn on their lamp after the study personnel leave the room. All packaging and labeling of the lamps will be performed by non-study personnel. Lamps will be assigned using a unique blind code which will be issued for each participant at the time of randomization. This ensures all individuals at the study centre remain blinded.

4.7 Study Population

Participants will be recruited from post-cardiac surgery patients experiencing moderate to severe depressive symptoms.

4.8 Inclusion and Exclusion Criteria

4.8.1 Inclusion Criteria

1) Undergoing cardiac surgery;
2) Age $\geq$ 18 years;
3) Score of $\geq$8 on the PHQ-9;
4) Able to understand and to provide informed consent in English or French; and
5) Likely to be available for follow-up.

4.8.2 Exclusion Criteria

1) Pregnant or lactating females;
2) Medical condition with a prognosis < 12 weeks;
3) History of bipolar disorder, SAD, psychosis, or dementia;
4) Medical condition contraindicating use of BLT (ocular or retinal pathology: glaucoma, cataracts, retinal detachment, retinopathy, diabetes);
5) Medical condition that increases photosensitivity (e.g. systemic lupus erythematosus,

rosacea, psoriasis, albinism);
6) Current use of medication that increases photosensitivity (e.g. St John's Wort, lithium, melatonin, tetracycline, Accutane, Benzoyl peroxide, Retin-A);
7) Less than one month following previous BLT;
8) Light induced epilepsy or migraines; or
9) Suicidal ideation.

5. TRIAL PROCEDURES

This study will be conducted according to ethical principles stated in the Declaration of Helsinki, and the consent forms will take into consideration the well-being, free-will and respect of the participants, including respect of privacy.

5.1 Recruitment

Participants will be recruited using recruitment flyers in the Cardiac Care Unit (CCU) and the cardiac surgery waiting room. Initial contact will be accomplished in person by the study coordinator prior to surgery. Interested potential participants will be asked to provide informed consent and confirm eligibility.

5.2 Enrollment

Informed consent will be obtained in person from all individuals. Potential participants will be given sufficient time to ask questions and decide whether or not to participate prior to signing and receiving a copy of the signed consent form. Study personnel will complete an inclusion and exclusion form to confirm eligibility based on the criteria listed above. As part of the screening process, potential participants will complete the PHQ-9 questionnaire to determine whether they are currently experiencing depressive symptoms. A cut off score of 8 will be used (sensitivity: 0.82; specificity: 0.83).[27] Potential participants who do not sign the consent form or who do not meet the eligibility criteria will be considered screen failures and will not be randomized to a treatment group.

5.3 Treatment Arms

5.3.1 Randomization to Treatment

Enrolled participants will be randomized (ratio of 1:1) at baseline using an online random number generator (Randomizer.org), to one of two treatments: (1) 10,000 lux BLT lamp, or (2) 500 lux modified BLT lamp. Randomization will be double-blinded, as neither the study personnel nor the participants will be aware of the treatment allocation. An information sheet will be provided to all participants to ensure that they are aware of the treatment plan and to provide the contact information of the study personnel. The 4 week treatment period will begin after surgery.

5.3.2 Treatment

Participants in the 10,000 lux lamp group will be supplied with an electric BLT lamp emitting 10,000 lux light intensity. Participants in the 500 lux lamp group will be supplied with an electric BLT lamp modified to emit 500 lux light intensity. All participants will be instructed to use the lamp for 30 minutes each morning, preferably upon waking, but not past 1:00 pm. Use will be monitored via self-

report at the day 4, day of discharge, and week 4 visits.

5.4 Follow-Up

5.4.1 Duration of Participation

Study participation begins once eligibility and consent have been established and continues until one of the following events occurs: (1) the week 12 questionnaires have been completed; (2) the participant withdraws his or her consent; or (3) the participant is lost-to-follow-up and the window for week 12 has closed. A study termination form will be completed for each participant when their study participation ends. Participants will complete a clinic visit on day 4, day of discharge, and at week 4, and will complete questionnaires via distance (online or by mail) at week 12.

5.4.2 Data Collection

A case report form (CRF) will be completed at baseline, day 4, day of discharge, and week 4. At week 12, participants will complete a survey to provide additional data. At baseline, the CRF will collect demographic and clinical information, including medical history, current medications, surgery details, and depression history. Participants will also complete three questionnaires: SF-36, PHQ-9, and DASS-21. At day 4, day of discharge, and week 4, participants will be asked about protocol adherence, symptoms, and AEs or SAEs. They will also complete the same three questionnaires to assess depressive symptoms and health-related quality of life. At week 12, participants will be asked to complete these same questionnaires as well as a survey assessing symptoms via distance either through mail or an online questionnaire website. During enrolment, participants will be asked about their preference (i.e. mail or online). If they choose the mail option, the questionnaires will be mailed to their address, along with a return envelope and stamp. If they choose the online option, they will be sent an email at week 12. The email will contain a link to the online survey (SurveyMonkey), which they can use to access the survey and complete the questionnaire.

5.4.3 Source Data

All data collected at baseline and follow-up visits on CRFs and questionnaires will be considered source data. Medical records will also be considered source data.

5.4.4 Schedule of Events

Visit	Window	Visit Type	CRF	Questionnaires
Baseline	N/A	In-Person	Yes	PHQ-9, SF-36, DASS-21
Day 4	± 1 day	In-Person	Yes	PHQ-9, SF-36, DASS-21
Day of discharge	± 1 day	In-Person	Yes	PHQ-9, SF-36, DASS-21

Week 4	± 7 days	In-Person	Yes	PHQ-9, SF-36, DASS-21
Week 12	± 7 days	Via Distance	No	PHQ-9, SF-36, DASS-21, survey assessing symptoms

5.4.5 Loss to Follow-up

Participants will be returning to the clinic for a post-operative medical appointment 4 weeks after their surgery. Their week 4 study visit will be scheduled for the same day, to help ensure participants' presence. At baseline, we will obtain contact information (email addresses, phone numbers, and addresses) for each participant as well as for at least one other individual who does not reside with the participant, to ensure that we will still be able to get in touch with participants if their phone numbers or addresses change. Finally, the commitment required for week 12 is minimal and participants will be compensated for their time. To minimize the amount of missing data due to loss to

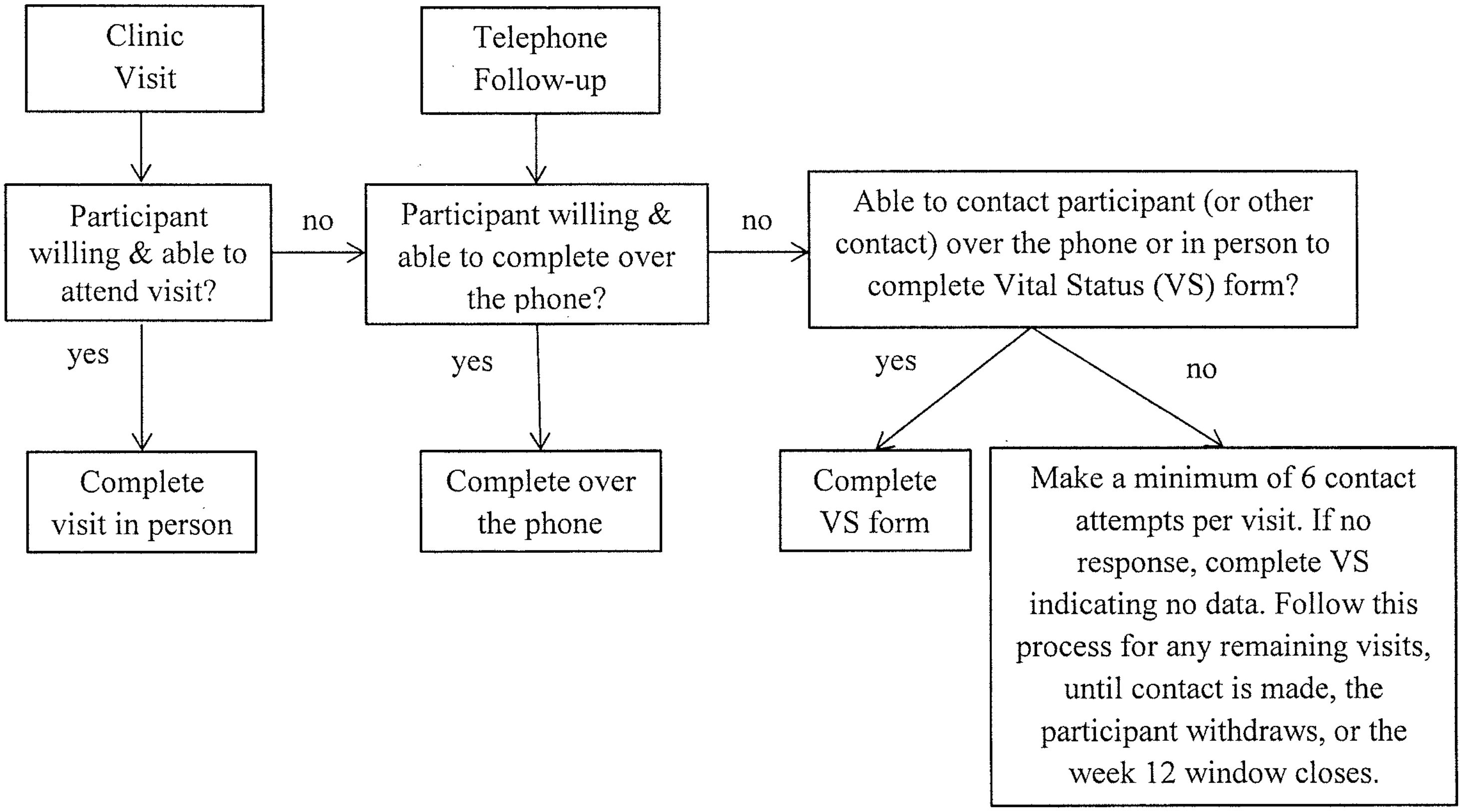

follow up, we will use the flow chart depicted below to collect as much data as possible for each participant.

5.4.6 Participant Withdrawal

Participants may withdraw at any time from the trial. A reason for withdrawal will be solicited from participants if possible. If the withdrawal is requested during the treatment period, participants will be requested to return the investigational product (lamp) to the study centre. No negative

consequences are anticipated from abrupt discontinuation of the investigational product. Data collected up to the point of withdrawal will be retained in the study database and used in analyses. Withdrawn participants will not be replaced.

5.4.7 Confidentiality

All information obtained from research participants will be treated confidentially within the limits of the law. No information that discloses the identity of participants will leave the enrolling institution. Other information will be coded and kept on a secure computer server at the Jewish General Hospital (Montreal, QC). All study information will be kept for a minimum of 25 years, under the responsibility of the principal investigator.

Unless specific authorization is provided by the participant, or where the law permits or a court order has been obtained, participant data will not be made available to third parties such as employers, governmental organizations, insurance companies or educational institutions. This also applies to the participant's spouse, other family members, or the participant's physician. However, for the purposes of ensuring the proper management of the research, a member of an ethics committee, a Health Canada representative or the study sponsor (Jewish General Hospital, Montreal QC) or its monitoring agents and representatives, may review participant research data as well as participant medical records for the purpose of monitoring this research.

6. TRIAL SAFETY

6.1 Collection of Safety Data

We will collect data regarding AEs, SAEs, and investigational product tolerability from participants at day 4, day of discharge, and weeks 4 and 12. All BLT lamps will be cleaned and sterilized before being given to participants, as well as when participants leave the hospital, to prevent possible spread of infection and reduce contamination concerns.

6.2 Adverse Events

An AE is any unfavourable and unintended sign, symptom, or disease temporally associated with the use of the trial product, whether or not considered related to the trial product. Study personnel will collect information concerning AEs at all follow-ups. Participants who experience AEs which may be related to the investigational product will be advised to contact study personnel concerning new or worsening symptoms. Study personnel will continue to assess AEs throughout the follow-up period.

6.3 Serious Adverse Events

A SAE is an AE that requires in-patient hospitalization or prolongation of existing hospitalization, that causes congenital malformation, that results in persistent or significant disability or incapacity, that is life threatening, or that results in death. Any other significant clinical event, not falling into the criteria above, but which in the opinion of the investigator requires reporting, may also be considered a SAE. If a SAE occurs, the investigator will gather any further documentation as needed (e.g., hospital discharge summaries, etc.). All SAEs will be reported to the Institutional Review Board (IRB). In the case of serious expected and/or unexpected suspected adverse reactions (SUSARs), Health Canada will be notified within 8 days of the event.

6.4 Unblinding

While unlikely, the investigator can decide whether or not to unblind a participant due to an AE or SAE. Participants can be unblinded by an authorized individual.

7. DATA ANALYSES

7.1 Description of Analyses

The primary analyses will describe the outcomes. The initial descriptive analysis will examine the balance of demographic variables between the 2 treatment groups. Due to randomization, known and unknown confounders should be equally distributed among the participants in the two treatment arms. Discrete data will be described using proportions. Continuous data will be described using means and standard deviations or, in the presence of skewed distributions, medians and inter-quartile ranges. These analyses will determine the recruitment rate, retention rate, protocol adherence rate, as well as process time needed. Finally, potential challenges, and important data values not collected will be assessed.

7.3 Sample Size Justification

The sample size of 38 participants (19 per arm) was chosen in order to appropriately test the study procedure and methods within the available timeframe. Acceptability and adherence to the intervention must be assessed before conducting a full trial. A sample size of 19 participants per arm should be able to demonstrate the ability to execute the specific protocol. If 100 eligible patients are identified, we will be able to estimate a participation rate of 38% to within a 95% Confidence Interval (CI) of +/- 7%. Additionally, with a sample size of 38, we will be able to estimate a compliance rate of 80% to within a 95% CI of +/- 8%.

REFERENCES

1. Garver D, Kaczmarek RG, Silverman BG, Gross TP, Hamilton PM. The epidemiology of prosthetic heart valves in the United States. Texas Heart Institute Journal 1995;22:86.
2. Huffman JC, Celano CM, Beach SR, Motiwala SR, Januzzi JL. Depression and cardiac disease: epidemiology, mechanisms, and diagnosis. Cardiovascular Psychiatry and Neurology 2013;2013.
3. Tully PJ, Baker RA. Depression, anxiety, and cardiac morbidity outcomes after coronary artery bypass surgery: a contemporary and practical review. Journal of Geriatric Cardiology : JGC 2012;9:197-208.
4. Pincus HA, Pettit AR. The societal costs of chronic major depression. The Journal of Clinical Psychiatry 2000;62:5-9.
5. Stewart WF, Ricci JA, Chee E, Hahn SR, Morganstein D. Cost of lost productive work time among US workers with depression. Jama 2003;289:3135-44.
6. Frasure-Smith N, Lespérance F, Gravel G, et al. Depression and health-care costs during the first year following myocardial infarction. Journal of psychosomatic research 2000;48:471-8.
7. Blumenthal JA. Depression and coronary heart disease: association and implications for treatment. Cleveland Clinic journal of medicine 2008;75 Suppl 2:S48-53.
8. Frasure-Smith N, Lespérance F, Talajic M. Depression and 18-month prognosis after myocardial infarction. Circulation 1995;91:999-1005.
9. Bush DE, Ziegelstein RC, Tayback M, et al. Even minimal symptoms of depression increase mortality risk after acute myocardial infarction. American Journal of Cardiology 2001;88:337-41.
10. Golden RN, Gaynes BN, Ekstrom RD, et al. The efficacy of light therapy in the treatment of mood disorders: a review and meta-analysis of the evidence. American Journal of Psychiatry 2005;162:656-62.
11. Lieverse R, Van Someren EJ, Nielen MM, Uitdehaag BM, Smit JH, Hoogendijk WJ. Bright light treatment in elderly patients with nonseasonal major depressive disorder: a randomized placebo-controlled trial. Archives of general psychiatry 2011;68:61-70.
12. Tuunainen A, Kripke DF, Endo T. Light therapy for non-seasonal depression. The Cochrane database of systematic reviews 2004:CD004050.
13. Kripke DF. Light treatment for nonseasonal depression: speed, efficacy, and combined treatment. Journal of affective disorders 1998;49:109-17.
14. Niederhofer H, von Klitzing K. Bright light treatment as mono-therapy of non-seasonal depression for 28 adolescents. International Journal of Psychiatry in Clinical Practice 2012;16:233-7.
15. Wirz-Justice A, Bader A, Frisch U, et al. A randomized, double-blind, placebo-controlled study of light therapy for antepartum depression. J Clin Psychiatry 2011;72:986-93.
16. Baxendale S, O'Sullivan J, Heaney D. Bright light therapy for symptoms of anxiety and depression in focal epilepsy: randomised controlled trial. The British Journal of Psychiatry 2013;202:352-6.
17. Kripke DF, Mullaney DJ, Klauber MR, Risch SC, Gillin JC. Controlled trial of bright light for nonseasonal major depressive disorders. Biological Psychiatry 1992;31:119-34.
18. Terman M, Terman JS. Light therapy for seasonal and nonseasonal depression: efficacy, protocol, safety, and side effects. CNS Spectrums 2005;10:647-63.
19. Shirani A, St. Louis EK. Illuminating Rationale and Uses for Light Therapy. Journal of Clinical Sleep Medicine : JCSM : Official Publication of the American Academy of Sleep Medicine 2009;5:155-63.
20. Kurlansik SL, Ibay AD. Seasonal affective disorder. Indian Jour of Clinical Practice 2013;24.
21. Terman M, Terman JS. Light therapy. Principles and Practice of Sleep Medicine 2005;4:1424-42.
22. Rastad C, Ulfberg J, Lindberg P. Improvement in fatigue, sleepiness, and health-related quality of

life with bright light treatment in persons with seasonal affective disorder and subsyndromal SAD. Depression Research and Treatment 2011.
23. Reeves GM, Nijjar GV, Langenberg P, et al. Improvement in depression scores after 1 hour of light therapy treatment in patients with seasonal affective disorder. The Journal of nervous and mental disease 2012;200:51-5.
24. Costco. Philips goLITE BLU. 2015.
25. Costco. Happylight Liberty Energy lamp with Replacement Bulb. 2015.
26. Naus T, Burger A, Malkoc A, Molendijk M, Haffmans J. Is there a difference in clinical efficacy of bright light therapy for different types of depression? A pilot study. Journal of affective disorders 2013;151:1135-7.
27. Manea L, Gilbody S, McMillan D. Optimal cut-off score for diagnosing depression with the Patient Health Questionnaire (PHQ-9): a meta-analysis. Canadian Medical Association Journal 2012;184:E191-E6.
